# Graph Theoretical Analysis of Structural Covariance Reveals the Relevance of Visuospatial and Attentional Areas in Essential Tremor Recovery After Stereotactic Radiosurgical Thalamotomy

**DOI:** 10.3389/fnagi.2022.873605

**Published:** 2022-05-23

**Authors:** Thomas A. W. Bolton, Dimitri Van De Ville, Jean Régis, Tatiana Witjas, Nadine Girard, Marc Levivier, Constantin Tuleasca

**Affiliations:** ^1^Department of Clinical Neurosciences, Neurosurgery Service and Gamma Knife Center, Centre Hospitalier Universitaire Vaudois (CHUV), Lausanne, Switzerland; ^2^Connectomics Laboratory, Department of Radiology, Centre Hospitalier Universitaire Vaudois (CHUV), Lausanne, Switzerland; ^3^Institute of Bioengineering, École Polytechnique Fédérale de Lausanne (EPFL), Lausanne, Switzerland; ^4^Department of Radiology and Medical Informatics, University of Geneva (UNIGE), Geneva, Switzerland; ^5^Stereotactic and Functional Neurosurgery Service and Gamma Knife Unit, Assistance Publique-Hôpitaux de Marseille, Centre Hospitalier Universitaire de la Timone, Marseille, France; ^6^Neurology Department, Assistance Publique-Hôpitaux de Marseille, Centre Hospitalier Universitaire de la Timone, Marseille, France; ^7^Department of Diagnostic and Interventional Neuroradiology, Centre de Résonance Magnétique Biologique et Médicale, Assistance Publique-Hôpitaux de Marseille, Centre Hospitalier Universitaire de la Timone, Marseille, France; ^8^Faculty of Biology and Medicine (FBM), University of Lausanne (UNIL), Lausanne, Switzerland; ^9^Signal Processing Laboratory (LTS5), École Polytechnique Fédérale de Lausanne (EPFL), Lausanne, Switzerland

**Keywords:** mean curvature, surface area, cortical thickness, graph theory, structural covariance analysis, stereotactic radiosurgical thalamotomy, radiosurgery, essential tremor

## Abstract

Essential tremor (ET) is the most common movement disorder. Its pathophysiology is only partially understood. Here, we leveraged graph theoretical analysis on structural covariance patterns quantified from morphometric estimates for cortical thickness, surface area, and mean curvature in patients with ET before and one year after (to account for delayed clinical effect) ventro-intermediate nucleus (Vim) stereotactic radiosurgical thalamotomy. We further contrasted the observed patterns with those from matched healthy controls (HCs). Significant group differences at the level of individual morphometric properties were specific to mean curvature and the post-/pre-thalamotomy contrast, evidencing brain plasticity at the level of the targeted left thalamus, and of low-level visual, high-level visuospatial and attentional areas implicated in the dorsal visual stream. The introduction of cross-correlational analysis across pairs of morphometric properties strengthened the presence of dorsal visual stream readjustments following thalamotomy, as cortical thickness in the right lingual gyrus, bilateral rostral middle frontal gyrus, and left pre-central gyrus was interrelated with mean curvature in the rest of the brain. Overall, our results position mean curvature as the most relevant morphometric feature to understand brain plasticity in drug-resistant ET patients following Vim thalamotomy. They also highlight the importance of examining not only individual features, but also their interactions, to gain insight into the routes of recovery following intervention.

## Introduction

Essential tremor (ET) is the most common movement disorder, affecting up to 5% of individuals above 65 years of age (Louis and Ferreira, 2010). Patients exhibit postural and kinetic tremor of the hands and arms, sometimes with head, legs, or voice tremor as well (Chunling and Zheng, 2016). Sensory deficiencies, cognitive deficits, psychiatric and sleep disorders can also complement motor symptoms (Chandran and Pal, 2012; Jhunjhunwala and Pal, 2014; Louis, 2016; Jiménez-Jiménez et al., 2020, 2021b). While ET has clear underlying genetic origins given the frequent occurrence of positive family history (Haubenberger and Hallett, 2018), the reliable identification of culprit genes remains partly unconclusive (Kuhlenbäumer et al., 2014; Tio and Tan, 2016; Deng et al., 2019; Siokas et al., 2020; Jiménez-Jiménez et al., 2021a).

Neuroimaging studies have enabled to localize the brain regions implicated in the motor component of ET, which belong to the so-called *tremor network* (Raethjen and Deuschl, 2012; Hallett, 2014; Sharifi et al., 2014): they include the cerebellum [sometimes regarded as the cornerstone of ET (Benito-León and Labiano-Fontcuberta, 2016; Ibrahim et al., 2020)], the motor thalamus (the ventro-intermediate nucleus, or Vim) and the motor cortex. While the exact pathophysiological mechanisms at play remain debated within several partly overlapping theories (Deuschl and Elble, 2009; Benito-Leon, 2014; Gironell, 2014), it is believed that ET manifests itself as a dysregulated network of interacting areas.

This makes the study of brain structure and function at the level of individual networks (to specifically address motor or non-motor impairments caused by ET, for instance), or at the whole-brain scale (to characterize cross-regional interactions in their entirety), a sensible analytical direction to pursue. Graph theory has emerged as the primary analytical approach for this purpose, since it provides an elegant and powerful way to gain insight into how information flows in complex systems described by *edges* linking the *vertices* of a graph.

Application areas are numerous: they include engineering problems such as the study of transportation systems (Derrible and Kennedy, 2011) or gear transmission (Xue et al., 2016), as well as life science disciplines such as proteomics (Grindrod and Kibble, 2004) or molecular topology (Amigó et al., 2009). Neuroscience has also been a particularly fruitful field for the application of graph theory (Bassett and Sporns, 2017; Sporns, 2018; Farahani et al., 2019); this is partly because a graph denoting the interplays between different brain regions can be meaningfully constructed from various imaging modalities, two notable examples of which are diffusion-weighted magnetic resonance imaging (DW-MRI, where edges are the physical connections between the areas) and resting-state functional magnetic resonance imaging (RS-fMRI, where edges denote the statistical dependencies between the activity time courses of the areas) (Bullmore and Sporns, 2009). More recent developments also include the generation of multilayer networks [e.g., interconnected temporally subsequent graphs capturing the dynamics of functional brain interactions (Preti et al., 2017; Pedersen et al., 2018)], and the subfields of graph signal processing, graph neural networks and graph learning (see Huang et al., 2018; Li R. et al., 2021 for recent reviews).

Graph theory has been pivotal in better grasping the neural underpinnings of cognition in the healthy and in the diseased brain (Minati et al., 2013; Hallquist and Hillary, 2018). ET has also been contemplated from this perspective: using DW-MRI and focusing on the executive network, Prasad et al. (2020) revealed lower global and local efficiencies of frontal executive brain centers and of the anterior cingulate cortex compared to matched healthy controls. In a conceptually similar study centered on the motor network, the local efficiency of the cerebellum was higher in ET patients, and those with resting tremor also showed further increases in the thalamus, globus pallidus, caudate, and supplementary motor area (Caligiuri et al., 2017).

Using RS-fMRI, Li J. Y. et al. (2021) evidenced lower nodal efficiency in frontal and supplementary motor areas, the precuneus and the cerebellum. Individuals with concomitant depression showed further pre-central, post-central, and frontal decreases. In another investigation, widespread differences that also involved other graph theoretical metrics (betweenness centrality, degree) were revealed in frontal, occipital, temporal, and cingulate cortices as well as subcortical and cerebellar loci (Benito-León et al., 2019).

From these reports, it transpires that ET-induced alterations of brain structure and function span multiple brain networks. In addition, these features of the disease can be captured by various graph theoretical measures. For these reasons, in the present work, we opted for studying ET through a whole-brain approach, and the combined extraction of a set of complementary graph theoretical metrics.

We propose three important advances compared to previous work: first, we study a well-defined population of drug-resistant patients with ET, who underwent Gamma Knife (GK) stereotactic radiosurgery of the Vim (Elaimy et al., 2010). We explore not only the differences between these subjects and matched healthy controls (HCs), but also brain plasticity 1 year after Vim thalamotomy. Only one past study has probed such mechanisms using graph theory (Jang et al., 2016), but the surgical intervention differed (imaging-guided high-intensity focused ultrasound thermal ablation). Moreover, the number of subjects was low (*N* = 10), and the analyses were centered on the motor network (with decreases in degree and efficiency following thalamotomy). Of note, the follow-up was interrupted 3 months after the intervention.

Second, we construct a graph neither from DW-MRI nor from RS-fMRI data as in the above cases, but from structural MR images. To do so, we leverage *structural covariance analysis* (SCA) (see Alexander-Bloch et al., 2013; Evans, 2013 for reviews), where the extent to which a morphometric measure of interest (e.g., cortical thickness) correlates across subjects in pairs of regions is used to build the graph. Such patterns of covariance are characteristic of the human cortex (Mechelli et al., 2005), are under genetic control (Schmitt et al., 2008; Romero-Garcia et al., 2018; Morgan et al., 2019), and recapitulate structural connectivity features (Yee et al., 2018). Here, we study three complementary features whose potential has extensively been validated (Bassett et al., 2008; Chen et al., 2008; He et al., 2008; Bernhardt et al., 2011; Bethlehem et al., 2017; Khundrakpam et al., 2017): cortical thickness (CT), surface area (SA), and mean curvature (MC). They encode partly unique information in the healthy brain (Chiarello et al., 2016), and undergo distinct environmental modulations (Kelly et al., 2013; Besteher et al., 2017), which justifies their parallel assessment.

To date, only one other work has applied graph theory to structural MR images in ET: Yang et al. (2021) quantified the similarity of gray matter profiles (Kullback–Leibler divergence-based) within regions to construct subject-wise graphs, and revealed altered metrics in frontal, temporal and angular gyri, the caudate, hippocampus, thalamus, and some parts of the cerebellum.

Third, in addition to the parallel assessment of individual morphometric properties, we propose a novel analysis that enables to additionally study cross-property dependences through the generation and analysis of directional graphs. This is motivated by the acknowledged genetic and phenotypic complementarity of the measures (Sanabria-Diaz et al., 2010; Yang et al., 2016), whose interactions may be altered by ET and/or thalamotomy. We introduce simple telling features that can be generated from such graphs, and interpret them in the context of our dataset.

## Materials and Methods

### Participants

We considered uniform structural MRI data from 34 ET patients (both before thalamotomy and one year later) scanned on the same 3T Siemens Skyra MR machine, and 29 HCs. All patients were right-sided and suffered from drug-resistant right-dominant ET. All underwent unilateral left Vim thalamotomy by GK.

The Timone University Hospital Ethical Committee (ID-RCB: 2017-A01249-44) granted formal approval for this study (including by the Ethics Committee at national level, CNIL-MR-03), and individual consent was also obtained from all subjects. Patients were neurologically evaluated and referred by TW, a neurologist specialized in movement disorders. All patients had a clear diagnosis of ET and showed no other structural abnormalities on pretherapeutic MRI. Demographic characteristics of the ET patients and HC subjects can be found in Table 1; both groups were matched for age and gender.

**TABLE 1:** Demographic and clinical details of the subjects.

Variable	HC	ET_*pre*_	ET_*post*_	Drop [points]	Drop [%]	*N*_*missing*_	*p*-value
***N***	29	34	34	n.a.	n.a.	n.a.	n.a.
**Age [years]**	69.93 ± 7.14 [59, 69, 83]	70.06 ± 9.12 [49, 72, 83]	n.a.	n.a.	n.a.	n.a.	*t_66_* = −0.06, *p* = 0.95
**Gender [M:F]**	12:17	17:17	17:17	n.a.	n.a.	n.a.	n.a.
**ADL**	n.a.	29.59 ± 11.39 [13, 28.5, 49]	6.03 ± 11.26 [0, 1, 41]	−23.56 ± 12.35 [−48, −24.5, 2]	82.83 ± 29.64 [0, 96.75, 100]	0/0	***t_66_* = 8.57, *p* = 2.48 10^–12^**
**HEAD**	n.a.	1 ± 0.85 [0, 1, 2]	0.56 ± 0.75 [0, 0, 3]	−0.39 ± 0.83 [−2, 0, 1]	n.a.	0/1	***t_65_* = 2.16, *p* = 0.035**
**QUEST**	n.a.	45.46 ± 16.4 [12, 41.5, 80]	23.16 ± 16.57 [1, 26, 57]	−24.79 ± 13.21 [−47, −25, −2]	n.a.	8/9	***t_43_* = 15.37, *p* = 4.47 10^–19^**
**TSTH**	n.a.	20.41 ± 5.53 [8, 20.5, 30]	6.26 ± 7.71 [0, 3, 27]	−14.15 ± 6.6 [−26, −14.5, 1]	72.73 ± 29.19 [0, 86.05, 100]	0/0	***t_66_* = 8.69, *p* = 1.52 10^–12^**
**Lesion volume [ml]**	n.a.	0.12 ± 0.13 [0.002, 0.076, 0.6]	n.a.	n.a.	n.a.	n.a.	n.a.
**Time to tremor arrest [days]**	n.a.	n.a.	127.56 ± 81.38 [15, 120, 300]	n.a.	n.a.	2	n.a.
**Symptoms duration [months]**	n.a.	35.53 ± 18.28 [5, 33, 61]	n.a.	n.a.	n.a.	n.a.	n.a.

*For healthy controls (HCs), patients before (ET_pre_) and after thalamotomy (ET_post_), values are reported as mean ± standard deviation, with minimum, median, and maximum into squared brackets. Some clinical scores could not be collected in a few occasions (N_missing_), in which case the associated subjects were excluded from statistical computations. Significant statistical comparisons are highlighted in bold. M, male; F, female.*

Several measures were used to clinically evaluate ET patients, and their recovery after the thalamotomy: Activities of Daily Living (ADL) from the survey designed by Bain et al. (1993), Tremor Score on Treated Hand (TSTH) from the Fahn–Tolosa–Marín rating scale (Fahn et al., 1993), head tremor (Tremor Research Group Essential Tremor Rating Assessment, from 0 to 3), and Quality of Life in Essential Tremor (QUEST) (Tröster et al., 2005). Clinical data is summarized in Table 1, where a significant improvement in clinical tremor scores can be observed across all the quantified measures upon thalamotomy.

Importantly, SCA is not compatible with the subject-wise investigation of these scores: indeed, only one measure of covariance is generated per group (e.g., post-therapeutic ET patients). Thus, one cannot conduct classical correlation analyses between morphometric features and clinical scores.

### Imaging

T1-weighted images were acquired on a head-only 3T machine (SIEMENS SKYRA, Munich, Germany, 32-channel receive-only phase-array head coil), with the following parameters: TR/TE = 2300/2.98 ms, isotropic voxels of 1 mm^3^, 160 slices.

As medication was frequently ineffective, most patients no longer received treatment at the time of Vim thalamotomy by GK. Thus, scanning was performed in a drug-naïve state (drugs having been stopped at least 3 days prior to scanning).

### Radiosurgical Procedure

Thalamotomy was performed with GK between September 2014 and April 2016, always at the Centre Hospitalier Universitaire de la Timone in Marseille. The surgeon in charge was JR, who used the Leksell Gamma Knife and associated Leksell GammaPlan software (Elekta Instruments, AB, Stockholm, Sweden). To avoid artifacts, DTI data was first acquired without the frame, and then co-registered with the therapeutic stereotactic images. The Leksell coordinate G frame (Elekta Instruments, AB, Stockholm, Sweden) was always applied under local anesthesia on the day of the thalamotomy. After positioning the frame, patients underwent both stereotactic CT and MRI.

Landmarks of interest, including the anterior and posterior commissures, were identified on an MR scan (particularly on T2 CISS/FIESTA sequence, replacing a former ventriculography). Uniform indirect targeting was performed using the Guiot diagram (Tuleasca et al., 2017), placed 2.5 mm above the anterior-posterior commissure line, and 11 mm lateral to the wall of the third ventricle. A single 4 mm isocenter was always used, and a maximum prescription dose of 130 Gy at the 100% isodose line was uniformly prescribed (Tuleasca et al., 2017).

### Computation of Morphometric Properties

The *Freesurfer* software (Fischl, 2012) was used to extract three morphometric measures of interest from structural MR images for a set of *P*_*C*_ = 68 cortical regions: CT, SA, and MC. Briefly, after linear registration to MNI space and bias field removal, the image at hand is skull-stripped (Ségonne et al., 2004), and voxels are classified as belonging to white matter or to another tissue category on the basis of their intensity and direct neighborhood. Hemispheres are separated, cerebellum and subcortex are removed, and the interface between the white and gray matters is located. From there, the pial surface is also tiled, and local estimates of CT, SA, and MC can be extracted (Fischl and Dale, 2000). Further details can be found in Dale et al. (1999) and Fischl et al. (1999). Eventually, local estimates are converted into *P*_*C*_ regional values per morphometric measure, using the Desikan-Killiany atlas (Desikan et al., 2006). These were complemented by measures of regional volume for *P*_*NC*_ = 19 non-cortical areas (including the brainstem, subcortical nuclei, and cerebellum), for a total of *P* = 87 parcels. They are summarized in Supplementary Table 1.

Obtained measurements were eventually linearly regressed out for age, gender, and total gray matter volume, separately within each group. The residuals were used for all subsequent analyses.

### Analysis of Individual Morphometric Measures

**FIGURE 1:**
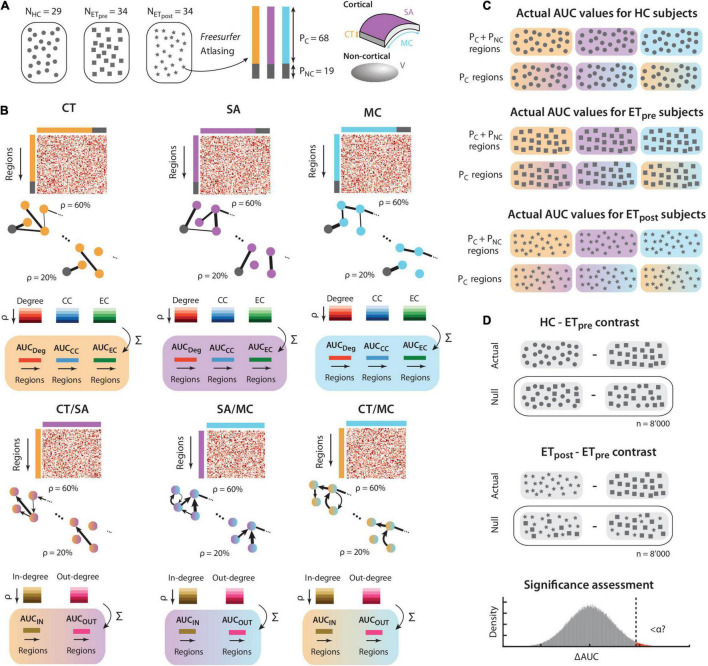
Schematic description of the method. **(A)** We consider three groups of subjects: healthy controls (*N* = 29, depicted by circles), and drug-resistant ET patients before and 1 year after Vim thalamotomy (*N* = 34, respectively denoted by rectangles and stars). For each structural scan, following *Freesurfer*-based processing and atlasing into regions of interest, the *P*_*C*_ = 68 cortical areas at hand can be described by their thickness (CT, orange), surface area (SA, purple), and mean curvature (MC, cyan). The *P*_*NC*_ = 19 non-cortical areas are characterized by their volume (V, gray). **(B)** Similarly for each of the three groups of subjects, cross-regional structural covariance is computed across subjects for CT (top left panel), SA (top middle panel), and MC (top right panel). The extracted information can equivalently be summarized in a matrix, or in a graph, where negative-valued structural covariance is set to zero. Regional degree, clustering coefficient (CC), and eigenvector centrality (EC) are computed for graph densities ranging from 20 to 60%, and the area under the curve (AUC) is taken as a regional output measure of interest for each morphometric property and graph metric case. In a similar fashion, cross-property covariance can be computed for the CT/SA (bottom left panel), SA/MC (bottom middle panel), and CT/MC (bottom right panel) cases. The obtained graphs are then directional, and in-degree and out-degree can be computed across graph densities to generate AUC output measures. **(C)** The computations described in panel **(B)** are performed similarly for the HC, ET_*pre*_, and ET_*post*_ groups. Note that cross-property metrics are only available for the *P*_*C*_ cortical regions. There is a total of 15 separate subcases with output AUC values: 3 morphometric properties (CT, SA, and MC) for 3 graph theoretical metrics (degree, CC, and EC), plus 3 cross-property pairs for 2 graph theoretical metrics (in-degree and out-degree). **(D)** Similarly for each of the 15 subcases at hand, the regional difference in AUC can be computed between the HC and ET_*pre*_ groups, or between the ET_*post*_ and ET_*pre*_ ones. The process described in panel **(B)** is then rerun *n* = 8’000 times after randomly shuffling the subjects across groups, to generate a null distribution of AUC differences. The actual value (vertical dashed line) is eventually compared to this null distribution to assess significance, with proper correction for the number of examined regions and subcases in parallel.

The process described below was identically conducted for each of the morphometric measures of interest. A schematic description of the undertaken steps, and of subsequent subparts of the analysis, is also provided in Figure 1.

Separately for the HC, pre-thalamotomy (abbreviated ET_*pre*_ from there onward) and post-thalamotomy (abbreviated ET_*post*_) data, Pearson’s correlation coefficient *R* was computed for each pair of regions. A positive/negative value means that when the measure in the first region is larger in one subject, it tends to be larger/lower in the second region.

To enable graph theoretical analysis, negative-valued edges were excluded (set to zero). The percentages of retained edges were: for CT, 79.6311% (HC group), 72.0663% (ET_*pre*_), 67.9497% (ET_*post*_); for SA, 67.3617% (HC), 69.1794% (HC_*pre*_), 71.8524% (ET_*post*_); for MC, 85.298% (HC), 77.2521% (ET_*pre*_), 81.5825% (ET_*post*_).

We computed graph theoretical measures from each structural covariance matrix to quantify regional properties of morphometric dependences with the rest of the brain. Here, we considered three different graph measures that convey complementary information: degree, clustering coefficient, and eigenvector centrality. Below, we briefly detail each, but more details can be found in Rubinov and Sporns (2010).

A group-wise structural covariance matrix can equivalently be seen as a graph *G*, characterized by an adjacency matrix **A**. In what follows, we generated a graph at a predefined density ρ (see below for more details about this parameter). We made sure that any graph analyzed therein was always fully connected.

Nodal degree for a region *p* is simply the sum of edge weights linked to the node at hand: $k_{p}=\sum_{i=1}^{P}A_{p,i}$. Larger values denote an overall more influential node within the network. The clustering coefficient is a measure of how much the direct neighborhood of a node is interconnected. The eigenvector centrality measures to what extent the node of interest takes part in modular communities.

To assess the differences between HC subjects and ET patients, we computed the difference HC – ET_*pre*_ for each regional graph metric. Similarly, to investigate the effects of thalamotomy, we computed the difference ET_*post*_ – ET_*pre*_.

Because the graph density at which to analyze the results is *a priori* unknown, we set to instead consider the area under the curve (AUC), or the sum of differences across densities ranging from 20% (the lowest value at which all graphs were fully connected) to 60% (for larger values, there would not be enough positive-valued SC elements).

For statistical assessment, these differences must be compared to an appropriate null distribution. To do so, we resorted to non-parametric permutation testing, by recomputing graph metrics over 8’000 null realizations for which subjects were randomly shuffled across groups.

False discovery rate (FDR)-corrected *p*-values were obtained and analyzed for each contrast of interest. When presenting our results, we considered two significance level α_1_ = 0.01 and α_2_ = 0.001.

### Cross-Property Analysis

In addition to traditional graph theoretical analysis, we sought to introduce another graph-centered approach to enable the investigation of cross-regional statistical dependences *across morphometric modalities*.

Consider the data from two morphometric properties, contained in **M**_1_ and **M**_2_ (each of size *P* x *S*, with *S* the number of subjects in the group at hand); the classical structural covariance equation would yield symmetrical matrices of size *P* x *P*: **SC**_1_ = cov(**M**_1_, **M**_1_)/σ_1_^2^ and SC_2_ = cov(**M**_2_, **M**_2_)/σ_2_^2^, with σ_*i*_ the standard deviation for property *i*.

Instead, we compute the cross-correlation **SC**_1,2_ = cov(**M**_1_, **M**_2_)/(σ_1_ σ_2_), which also has size *P* x *P*, but is not symmetrical anymore. In this matrix, if element (*i*,*j*) is positive/negative, then when modality 1 in region *i* is larger in a given subject, modality 2 in region *j* will tend to be larger/lower. However, if element (*j*,*i*) is positive/negative, then when modality 1 in region *j* is larger in a given subject, then modality 2 in region *i* will be larger/lower.

Let CT, SA, and MC be respectively denoted by the indices 1, 2, and 3; we can thus compute three cross-correlation matrices: **SC**_1,2_, **SC**_1,3_, and **SC**_2,3_. Each can equivalently be seen as a directional graph (*G*_1,2_, *G*_1,3_, and *G*_2,3_), or a non-symmetrical adjacency matrix (**A**_1,2_, **A**_1,3_, and **A**_2,3_). For simplicity, we set null diagonal entries to exclude self-loops, and exclude negative-valued edges.

Here, with inspiration from time-resolved functional neuroimaging work on directional graphs (Bolton et al., 2020), we propose to compute two simple measures from each graph: (1) the in-degree, and (2) the out-degree. The in-degree for modality pair (*m*_1_, *m*_2_) at region *j* is defined as $k_{\text{IN},j}=\sum_{i=1}^{P_{C}}A_{m_{1},m_{2}}\,\left(i,j\right)$, and the out-degree as $k_{\text{OUT},j}=\sum_{i=1}^{P_{C}}A_{m_{1},m_{2}}\,\left(j,i\right)$. The in-degree is larger if modality *m*_2_ in region *j* is more strongly dependent on the values of modality *m*_1_ in the other brain areas. The out-degree is larger if modality *m*_1_ in region *j* more strongly influences the values of modality *m*_2_ in the other brain areas.

We resort to the same non-parametric approach as above for statistical significance assessment. In more details, for both metrics and for each of the three pair-wise modality cases, we compute the HC – ET_*pre*_ and ET_*post*_ – ET_*pre*_ group differences and compare them to a null distribution generated following random shuffling of the subjects across groups (*n* = 8’000 permutations).

### Availability of the Data and Scripts

All the analytical steps described above were performed with custom scripts and MATLAB2014b (MathWorks, Natick, MA, United States). Colormaps for plotting were generated with the *cbrewer* toolbox.^1^ Investigated graph measures were computed using the *Brain Connectivity Toolbox* (Rubinov and Sporns, 2010).

Data sharing is not applicable to this article as no new data were created or analyzed in this study. All the scripts used in this work are freely available at https://github.com/TiBiUan/SCA_GraphTheoretical.git.

## Results

The results of the analyses centered on CT are presented in Supplementary Figure 1. None of the probed graph theoretical metrics yielded any significant outcome following FDR correction. For degree (Supplementary Figure 1A) and eigenvector centrality (Supplementary Figure 1C), smaller metric values in the ET_*post*_ than in the ET_*pre*_ group in the left parahippocampal gyrus was nonetheless a noteworthy, shared feature, with the actual group difference falling within the bulk of null distribution outliers. For clustering coefficient (Supplementary Figure 1B), ET_*pre*_ values were smaller than HC ones in the brainstem, but the smallest edge density cases were by far the largest contributors, as denoted by the dominance of the dark blue shades in the HC and ET_*post*_ stacked bars.

The results of surface area analyses are presented in Supplementary Figure 2. None of the investigated cases reached significance. As with cortical thickness, the left parahippocampal gyrus degree (Supplementary Figure 2A) and eigenvector centrality (Supplementary Figure 2C) values were nonetheless noteworthily larger in the ET_*pre*_ group compared to the ET_*post*_ one. Clustering coefficient results (Supplementary Figure 2B) were overall very similar across all groups.

**FIGURE 2:**
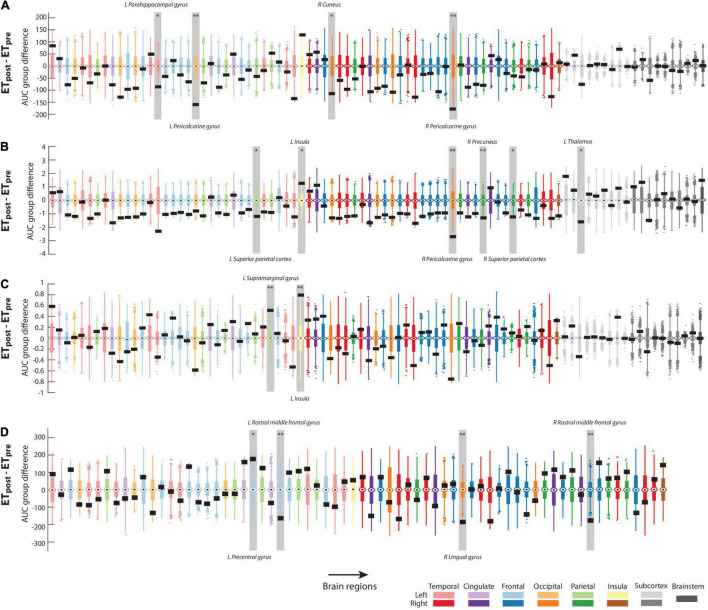
Significant graph theoretical analysis findings. For regional degree **(A)**, clustering coefficient **(B)**, eigenvector centrality **(C)**, and out-degree **(D)**, group differences in area under the curve for the ET_*post*_ – ET_*pre*_ contrast in the assessment of mean curvature structural covariance **(A–C)** or cortical thickness/mean curvature interactions **(D)**. Actual values are denoted by black rectangles, and associated null distributions are reflected by box plots whose color coding matches the brain lobe at hand. The regions that reached significance in each case are highlighted by a light gray box and labeled. *: *p* < 0.01; ^**^: *p* < 0.001.

Figures 2A–C displays the results from mean curvature analyses (a more exhaustive version is also available as Supplementary Figure 3). For degree (Figure 2A) and the ET_*post*_ – ET_*pre*_ contrast, values were significantly larger pre-intervention in the left parahippocampal gyrus (region 15, Δ_*AUC*_ = −86.9766, *p* = 0.01), bilateral pericalcarine gyrus [regions 20 and 54, Δ_*AUC*_ = −159.3861 and −178.5003 (left and right sides, respectively), *p* < 0.001 in both cases], and right cuneus (region 38, Δ_*AUC*_ = −114.0338, *p* < 0.01). For clustering coefficient (Figure 2B), 6 regions reached significance when assessing the impacts of intervention: values were larger before thalamotomy in the bilateral superior parietal cortex [regions 28 and 62, Δ_*AUC*_ = −1.1811 and −1.2203 (left and right sides, respectively), *p* < 0.01 in both cases], right pericalcarine gyrus (region 54, Δ_*AUC*_ = −2.6884, *p* < 0.001), right precuneus (region 58, Δ_*AUC*_ = −1.3199, *p* < 0.001), and left thalamus (region 71, Δ_*AUC*_ = −1.6116, *p* < 0.01). Conversely, clustering coefficient increased after the intervention in the left insula (region 34, Δ_*AUC*_ = 1.2759, *p* < 0.01). For eigenvector centrality (Figure 2C), the left supramarginal gyrus and left insula showed significantly larger values after the intervention (respectively regions 30 and 34, Δ_*AUC*_ = 0.5126 and 0.7964, *p* < 0.001 in both cases).

Regarding the interaction between morphometric properties, there were no significant results involving surface area and any of the two other features (Supplementary Figures 4, 5). Regarding the cortical thickness/mean curvature case (Figure 2D; see also Supplementary Figure 6 for more exhaustive displays), the out-degree was significantly larger in the ET_*post*_ than ET_*pre*_ group in the left pre-central gyrus (region 23, Δ_*AUC*_ = 178, *p* < 0.01), and lower in the right lingual gyrus (region 46, Δ_*AUC*_ = −185, *p* < 0.001) and bilateral rostral middle frontal gyrus [regions 26 and 60, Δ_*AUC*_ = −163 and −177 (left and right sides, respectively), *p* < 0.001 in both cases].

## Discussion

In the present work, we leveraged graph theoretical analysis to study structural covariance patterns in patients with ET before and after thalamotomy, as well as compared to age-matched HCs. In doing so, we considered three popular and complementary morphometric properties: cortical thickness, surface area, and mean curvature. All the significant results from our analyses are summarized in Table 2.

**TABLE 2:** Summary of significant results.

Region	Morphometric property	Graph metric	Contrast	Δ AUC	*p*-value
L Parahippocampal Gyrus	MC	D	ET_*post*_ – ET_*pre*_	−86.9766	<0.01
**L Pericalcarine Gyrus**	**MC**	**D**	**ET_*post*_** –** ET_*pre*_**	**−159.3861**	**<0.001**
**R Pericalcarine Gyrus**	**MC**	**D**	**ET_*post*_** –** ET_*pre*_**	**−178.5003**	**<0.001**
R Cuneus	MC	D	ET_*post*_ – ET_*pre*_	−114.0338	<0.01
L Superior Parietal Cortex	MC	CC	ET_*post*_ – ET_*pre*_	−1.1811	<0.01
R Superior Parietal Cortex	MC	CC	ET_*post*_ – ET_*pre*_	−1.2203	<0.01
**R Pericalcarine Gyrus**	**MC**	**CC**	**ET_*post*_** –** ET_*pre*_**	**−2.6884**	**<0.001**
**R Precuneus**	**MC**	**CC**	**ET_*post*_** –** ET_*pre*_**	**−1.3199**	**<0.001**
L Thalamus	MC	CC	ET_*post*_ – ET_*pre*_	−1.6116	<0.01
L Insula	MC	CC	ET_*post*_ – ET_*pre*_	1.2759	<0.01
**L Supramarginal Gyrus**	**MC**	**EC**	**ET_*post*_** –** ET_*pre*_**	**0.5126**	**<0.001**
**L Insula**	**MC**	**EC**	**ET_*post*_** –** ET_*pre*_**	**0.7964**	**<0.001**
L Pre-central Gyrus	CT → MC	Out-degree	ET_*post*_ – ET_*pre*_	178	<0.01
**R Lingual Gyrus**	**CT** → **MC**	**Out-degree**	**ET_*post*_** –** ET_*pre*_**	**−185**	**<0.001**
**L Rostral Middle Frontal Gyrus**	**CT** → **MC**	**Out-degree**	**ET_*post*_** –** ET_*pre*_**	**−163**	**<0.001**
**R Rostral Middle Frontal Gyrus**	**CT** → **MC**	**Out-degree**	**ET_*post*_** –** ET_*pre*_**	**−177**	**<0.001**

*Regions associated to significant results are listed alongside the morphometric property at hand (MC, mean curvature; CT, cortical thickness), the graph metric for which the result was found (D, degree; CC, clustering coefficient; EC, eigenvector centrality), the contrast that yielded the group difference, the associated difference in area under the curve (ΔAUC) and false discovery rate-corrected p-value. Regions significant at the more stringent threshold of p < 0.001 are highlighted in bold.*

Interestingly, the comparison between pre-thalamotomy patients with ET and HCs did not yield any significant outcome. There are several potentially overlapping explanations to this negative finding. First, the assessment of structural covariance may be less useful than other approaches (such as DW-MRI- or RS-fMRI-based ones) to unravel the brain alterations induced by ET due to its less specific nature. Second, it could be that graph theoretical analysis is not the suited tool to unravel ET-related SC alterations; for instance, the finer-grained assessment of individual cross-regional interactions (as opposed to more global summarizing metrics as considered here) may be a more fruitful research avenue that should be explored in future work. Third, the anatomical underpinnings of ET may bear a subject-specific nature, and hence, may not be adequately captured by a group-level analysis relying on correlations across subjects as deployed here. Along this line of reasoning, it is interesting to notice that essential tremor is, nowadays, considered by many as a family of disparate diseases rather than a single entity (Jankovic, 2002; Elble, 2013; Espay et al., 2017; Louis, 2021).

One could hypothesize that thalamotomy, by renormalizing morphometric features, yields a more homogeneous group compared to HCs or ET patients before intervention. Accordingly, we found several significant impacts of Vim stereotactic radiosurgical thalamotomy. First, mean curvature was by far the most impacted morphometric property following the intervention. This implies that mechanisms of brain plasticity may occur more easily regarding this geometric aspect of the brain. Cortical gyrification is a genetically regulated developmental process (Papini et al., 2020), which is also specifically altered over other morphometric properties in schizophrenia (Schultz et al., 2010, 2013). Interestingly, mean curvature can be affected by experience alone: for example, gyrification weakens less rapidly upon aging in bilinguals (Del Maschio et al., 2019), and meditation practitioners show a greater extent of gyrification in several regions of the brain compared to matched non-practitioners (Luders et al., 2012). In a study following patients suffering from anorexia nervosa, Bernardoni et al. (2018) also found that cortical folding was broadly lowered, but that weight restoration within only 3 months sufficed to restore the values to a normal level. Taken together, these reports lend credit to the possibility that within one year after Vim thalamotomy, mean curvature could indeed have underwent significant changes in patients with ET, without as extensive modulations of other geometrical brain features.

Another factor that may have contributed to the presence of extensive differences in the ET_*post*_ – ET_*pre*_, but not the HC – ET_*pre*_ contrast case, is the assessment of the same subjects in both groups for the former, but not the latter comparison. Indeed, our analyses did not explicitly model within-subject variance, because the resulting graphs then bear a different interpretation (see Supplementary Material for details). However, we explicitly verified that with a mixed model approach accounting for repeated measures, whole-brain ET_*post*_ – ET_*pre*_ structural covariance difference patterns remain largely identical to those analyzed therein (see Supplementary Figure 7). The only morphometric property for which there were some noticeable impacts was SA, for which structural covariance differences took a narrower range when modeling within-subject variance; recall that these differences were already non-significant in our analyses. For MC, the match across methods was the largest (spatial correlation of whole-brain patterns: 0.97), leading us to rule out a major impact of repeated measures on our findings.

Turning to the areas involved in the observed differences, the left thalamic clustering coefficient was larger before the intervention (at our less stringent significance threshold of *p* < 0.01), revealing that one of its consequences was the loss of cross-regional dependences within its neighboring areas.

Collectively, our other findings point at important rearrangements revolving around the dorsal visual pathway, which is involved in visuospatial attention and action guidance (Freud et al., 2016). First, several low-level visual areas (bilateral pericalcarine gyrus, and to a lesser extent, also the right cuneus) showed a lower degree following thalamotomy.

Second, higher-level regions in the dorsal visual stream also exhibited mean curvature changes upon intervention: the left parahippocampal gyrus, linked to visuospatial processing (Aminoff et al., 2013), displayed lower degree at our more lenient significance threshold; the left supramarginal gyrus, involved in the planning of visually guided reaching and grasping movements (Andres et al., 2017; McDowell et al., 2018; Potok et al., 2019), exhibited a larger eigenvector centrality; in addition, clustering coefficient was significantly smaller (at *p* < 0.01) in the bilateral superior parietal cortex, a cornerstone region bridging visual and motor functions (Corbetta et al., 1995; Husain and Parashkev, 2007), as well as in the right precuneus, directly tied to visuospatial attention in past transcranial magnetic stimulation work (Mahayana et al., 2014) and known to jointly functionally relate to motor, visual and cognitive brain centers (Margulies et al., 2009).

Third, the eigenvector centrality of the left insula [classically known for its role in salience monitoring (Menon and Uddin, 2010), and directly associated to attentional performance (Varjačić et al., 2018)] also increased post-intervention.

Beyond the individual assessment of each morphometric property, our novel cross-feature analysis also enabled to evidence the presence of Vim thalamotomy-induced changes in the brain that modify the interplay between CT and MC. Out-degree was lower after intervention in the right lingual gyrus; in other words, this region’s cortical thickness is normally statistically related to overall mean curvature in the rest of the brain, and the dependence was lowered upon thalamotomy. A similar observation was made concerning the bilateral rostral middle frontal gyrus, which is a sub-component of the dorsolateral prefrontal cortex, an area involved in attentional functions (Kondo et al., 2004; Johnson et al., 2007). These two findings show that modulations at the level of the dorsal visual stream also extend to dependences between two different morphometric properties. Finally, out-degree increased (albeit with only mild significance) in the left pre-central gyrus, home of the motor cortex contralateral to the targeted side; this may reflect compensatory brain plasticity, where left pre-central cortical thickness gains new relationships to the mean curvature of other brain regions.

The presence of interactions across morphometric properties is unsurprising given the joint orchestration of their patterns upon brain development (Raznahan et al., 2011; Schnack et al., 2015); our study calls for further efforts to not only analyze a given morphometric feature independently, but to consider all of them jointly for a more complete understanding of the brain. In that, our cross-modality analysis strategy somewhat echoes recent work in which a handful of modalities are jointly analyzed to generate subject-wise *morphometric similarity networks* (Seidlitz et al., 2018). The advantage of this approach over ours is that measures can be estimated for each individual, enabling to then readily probe possible relationships to clinical scores. Its drawback may lie in the interpretability of the results; by instead solely addressing the dependence between two measures, mechanistic insight may be easier to achieve.

Our study bears some important limitations that should be kept in mind: in particular, the number of subjects remains quite low. This is largely because the analyzed data was collected at two successive time points with a one-year gap. Our results should thus be considered with caution, in the specific setting of the dataset that we analyzed. Further work on a larger pool of subjects will be required to determine whether the findings presented therein can generalize.

Another limitation is the use of a somewhat spatially gross atlas compared to state-of-the-art (sub)cortical alternatives (Schaefer et al., 2018; Tian et al., 2020). While this certainly precludes our ability to more finely capture ET-induced alterations and their evolution upon intervention, the number of statistical tests conducted in parallel would dramatically increase with a finer-grained parcelation, and so would the extent of dependences between spatially neighboring areas. On top of rendering interpretation much more complex, tailored statistical correction methods would then have to be deployed for appropriate analysis.

Partly linked to the above point is the fact that, while most pinpointed areas could be related to visual or attentional functions (13 of 16 or 8 of 9 as a function of the considered significance threshold), their relatively large size makes it possible that the observed thalamotomy-induced changes instead associate to other functions subserved by neighboring foci.

Aside from graph theoretical approaches, there exist many other ways by which structural covariance can be probed. Alongside the route undertaken here, an alternative option for future work could be to investigate in greater details the specificities of whole-brain structural covariance with specific regions of interest. For example, a seed-based structural covariance map can be computed in each group at stake, and measures such as their spatial properties can be compared (DuPre and Spreng, 2017). Another alternative is the use of Partial Least Squares analysis to generate seed-to-whole brain covariance maps to then contrast across groups of interest (Persson et al., 2014).

Yet another main analytical line involves the decomposition of the morphometric data into different summarizing components, as can be achieved using various approaches such as Independent Component Analysis (Hafkemeijer et al., 2016), Principal Component Analysis (Brickman et al., 2008; Bergfield et al., 2010), non-negative matrix factorization (Sotiras et al., 2015, 2017), or diffusion embedding (Masouleh et al., 2020). In this study, we decided not to conduct such analyses because they work optimally when the data has a voxel-wise spatial resolution, whereas we addressed regional properties.

Finally, other more recent approaches can also be envisaged: in particular, the *person-based similarity index* (PBSI) is a recent measure that quantifies how similar a given subject is to all the others in terms of its whole-brain morphometric profile (Doucet et al., 2019, 2020) and can be directly applied to regional data. In future work, it will be interesting to see whether such tools enable to go beyond the group-level characterization, to start gaining further insight into individual specificities, and what leads some subjects to recover from ET more effectively than others upon surgery.

## Conclusion

In this work, we evidenced a significant impact of Vim thalamotomy on cortical gyrification in drug-resistant ET patients. Changes following the intervention could be captured, across complementary graph theoretical metrics, within visuospatial and attentional areas. This denotes the network-level reconfiguration of these regions’ statistical dependencies to the rest of the brain, and calls for more extensive investigations of the visual circuitry in future studies of ET.

## Data Availability Statement

The raw data supporting the conclusions of this article will be made available by the authors, without undue reservation.

## Ethics Statement

The studies involving human participants were reviewed and approved by the Timone University Hospital Ethical Committee. The patients/participants provided their written informed consent to participate in this study.

## Author Contributions

TB wrote the scripts, performed the analyses, and wrote the original manuscript draft. JR, TW, and NG contributed to data acquisition. DV and ML surveyed the work, provided guidance regarding the analyses, and helped interpret the results. CT contributed to data processing as well as the analysis and interpretation of the results. All authors thoroughly reread the manuscript.

## Conflict of Interest

The authors declare that the research was conducted in the absence of any commercial or financial relationships that could be construed as a potential conflict of interest.

## Publisher’s Note

All claims expressed in this article are solely those of the authors and do not necessarily represent those of their affiliated organizations, or those of the publisher, the editors and the reviewers. Any product that may be evaluated in this article, or claim that may be made by its manufacturer, is not guaranteed or endorsed by the publisher.

## Footnotes

1
https://www.mathworks.com/matlabcentral/fileexchange/34087-cbrewer-colorbrewer-schemes-for-matlab
