# Supplementary material for: Graph Theoretical Analysis of Structural Covariance Reveals the Relevance of Visuospatial and Attentional Areas in Essential Tremor Recovery After Stereotactic Radiosurgical Thalamotomy
Source: Front Aging Neurosci. 2022 May 23;14:873605. doi: 10.3389/fnagi.2022.873605 (PMC9168220; doi:10.3389/fnagi.2022.873605)
Supplement: Supplementary file 1 [file Data_Sheet_1.pdf]

## Supplementary analysis: mixed model approach for the $ET_{post}$ – $ET_{pre}$ contrast

In order to assess whether explicitly accounting for within-subject variance may impact the outcomes from structural covariance analysis in the longitudinal $ET_{post}$ - $ET_{pre}$ case, we conducted a supplementary investigation in which a mixed model approach was implemented instead of the original Pearson's correlation-based computations.

As a reminder, in the structural covariance assessments presented in the main body of this work, cross-regional correlation across subjects for a morphometric measure of interest was computed separately for each group, *via* Pearson's correlation coefficient: in each region pair case, this thus yielded one output value for the $ET_{pre}$ group, and one for the $ET_{post}$ group. Computations were performed on demeaned morphometric data regressed out for age, gender and total grey matter volume. Graph theoretical metrics (degree, clustering coefficient and eigenvector centrality) were then computed from the $ET_{pre}$ and the $ET_{post}$ graphs at various edge densities, focusing on positive-valued edges. For each graph metric at hand, the difference $ET_{post}$ - $ET_{pre}$ was summed across the investigated densities, and for significance assessment, this statistic was eventually compared to a null distribution non-parametrically generated upon random shuffling of subjects across groups.

In order to account for the fact that two measures (one before and one 1 year after intervention) were available per subject, a mixed model including a random effect term for subjects can be considered. For this purpose, the covariance between regions *i* and *j* for a given morphometric measure could, in the simplest form, be expressed in terms of an output variable (the morphometric value for region *j*) and a set of predictors (including the value for region *i*, age, gender, and total grey matter volume). The model would take the form:

$$M_{i,s} = \beta_0 + \beta_1 M_{j,s} + \beta_2 A_s + \beta_3 GMV_s + \beta_4 G_s + b_{0,s} + \varepsilon_{i,s}. \quad (1)$$

In the above equation, $M_{i,s}$ denotes the morphometric measure at hand for region $i$ and subject $s$, $\beta_0$ is the model's intercept, $\beta_1$ would be the equivalent of structural covariance between the regions, $A_s$, $GMV_s$ and $G_s$ denote the age, total grey matter volume and gender of subject $s$ and are respectively associated to the $\beta_2$, $\beta_3$ and $\beta_4$ coefficients. The term $b_{0,s}$ is the random effect for subject $s$ (that is, a subject-wise constant contributing to the estimation of $M_{i,s}$), and follows a normal distribution with mean 0 and standard deviation $\sigma_b$. The term $\varepsilon_{i,s}$ denotes the error for region $i$ and subject $s$, and also follows a normal distribution with mean 0 and standard deviation $\sigma_\varepsilon$. Since structural covariance was quantified through Pearson's correlation coefficient in our original analyses, note that in this equation, the morphometric measures in each group have been normalized by their standard deviation across subjects.

If one were to fit such a model once for the $ET_{pre}$ group and once for the $ET_{post}$ group, in order to match our original analyses, *there would only be one measure per subject per model*. In such a setting, the modelling of random effects thus does not bring any added value to the problem. In order to benefit from the inclusion of a random effect term, one needs to instead directly model the $ET_{post}$ - $ET_{pre}$ contrast. The model then takes the form:

$$M_{i,s} = \beta_0 + \beta_1 M_{j,s} + \beta_2 \Delta_s + \beta_3 \Delta_s M_{j,s} + \beta_4 A_s + \beta_5 GMV_s + \beta_6 G_s + b_{0,s} + \varepsilon_{i,s}. \quad (2)$$

The morphometric measure of interest in region $i$ for subject $s$ now depends on the interaction between the morphometric measure in region $j$ and the contrast sign $\Delta_s$ for subject $s$. The coefficient of interest in this case is that of the interaction ($\beta_3$), which directly quantifies the *difference in structural covariance* between the $ET_{pre}$ and $ET_{post}$ groups.

While the inclusion of a random effect term is then informative, the estimated quantity cannot be readily matched to that of our original graph theoretical pipeline. This is because each of the extracted edges reflects a group difference, whereas in our original analyses, we first quantified graph theoretical metrics, and only then derived the $ET_{post}$ – $ET_{pre}$ group difference.

The graph that is generated through mixed modelling conceptually differs from the ones that we originally handled: for example, the sign of an edge now reflects the direction of the assessed group difference, as opposed to the strength of structural covariance. Graph theoretical metrics, if computed, would thus not lend themselves to the same interpretation.

Notwithstanding the conceptual differences between the quantities obtained through both analytical strategies, we assessed the impact of including a random effect term on the results. To do so, we implemented the mixed model strategy for the $ET_{post}$ - $ET_{pre}$ group difference, and compared the obtained whole-brain structural covariance difference pattern to the one yielded by our original approach, when computing edge-wise structural covariance separately in each group and then quantifying the $ET_{post}$ - $ET_{pre}$ contrast. For cortical thickness, surface area and mean curvature, Pearson's correlation between the resulting whole-brain patterns was respectively equal to 0.94, 0.88 and 0.97. As confirmed by **Supplementary Figure 7**, the extracted patterns with both approaches are extremely similar, with the only noticeable difference for surface area, where the range of values obtained using the mixed model approach is narrower. For mean curvature, which yielded all significant findings in our original analyses, correlation was the highest. Thus, the modelling of within-subject variance only has minimal impacts on obtained structural covariance results.

**Supplementary Table 1: Regions of the considered atlas.** All the regions analyzed in this work are listed alongside their index, and the hemisphere to which they belong.

| **Index** | **Hemisphere** | **Name** |
|---|---|---|
| 1 | Left | Banks superior temporal sulcus |
| 2 | Left | Caudal anterior cingulate cortex |
| 3 | Left | Caudal middle frontal cortex |
| 4 | Left | Cuneus |
| 5 | Left | Entorhinal cortex |
| 6 | Left | Fusiform gyrus |
| 7 | Left | Inferior parietal cortex |
| 8 | Left | Inferior temporal cortex |
| 9 | Left | Isthmus cingulate |
| 10 | Left | Lateral occipital cortex |
| 11 | Left | Lateral orbitofrontal cortex |
| 12 | Left | Lingual cortex |
| 13 | Left | Medial orbitofrontal cortex |
| 14 | Left | Middle temporal cortex |
| 15 | Left | Parahippocampal gyrus |
| 16 | Left | Paracentral gyrus |
| 17 | Left | Pars opercularis |
| 18 | Left | Pars orbitalis |
| 19 | Left | Pars triangularis |
| 20 | Left | Pericalcarine gyrus |
| 21 | Left | Postcentral gyrus |
| 22 | Left | Posterior cingulate cortex |
| 23 | Left | Precentral gyrus |
| 24 | Left | Precuneus |
| 25 | Left | Rostral anterior cingulate cortex |
| 26 | Left | Rostral middle frontal cortex |
| 27 | Left | Superior frontal cortex |
| 28 | Left | Superior parietal cortex |
| 29 | Left | Superior temporal cortex |
| 30 | Left | Supramarginal gyrus |
| 31 | Left | Frontal pole |
| 32 | Left | Temporal pole |
| 33 | Left | Transverse temporal cortex |

| | | |
|---|---|---|
| 34 | Left | Insula |
| 35 | Right | Banks superior temporal sulcus |
| 36 | Right | Caudal anterior cingulate cortex |
| 37 | Right | Caudal middle frontal cortex |
| 38 | Right | Cuneus |
| 39 | Right | Entorhinal cortex |
| 40 | Right | Fusiform gyrus |
| 41 | Right | Inferior parietal cortex |
| 42 | Right | Inferior temporal cortex |
| 43 | Right | Isthmus cingulate |
| 44 | Right | Lateral occipital cortex |
| 45 | Right | Lateral orbitofrontal cortex |
| 46 | Right | Lingual cortex |
| 47 | Right | Medial orbitofrontal cortex |
| 48 | Right | Middle temporal cortex |
| 49 | Right | Parahippocampal gyrus |
| 50 | Right | Paracentral gyrus |
| 51 | Right | Pars opercularis |
| 52 | Right | Pars orbitalis |
| 53 | Right | Pars triangularis |
| 54 | Right | Pericalcarine gyrus |
| 55 | Right | Postcentral gyrus |
| 56 | Right | Posterior cingulate cortex |
| 57 | Right | Precentral gyrus |
| 58 | Right | Precuneus |
| 59 | Right | Rostral anterior cingulate cortex |
| 60 | Right | Rostral middle frontal cortex |
| 61 | Right | Superior frontal cortex |
| 62 | Right | Superior parietal cortex |
| 63 | Right | Superior temporal cortex |
| 64 | Right | Supramarginal gyrus |
| 65 | Right | Frontal pole |
| 66 | Right | Temporal pole |
| 67 | Right | Transverse temporal cortex |
| 68 | Right | Insula |
| 69 | Left | Cerebellar white matter |
| 70 | Left | Cerebellum |

| | | |
|---|---|---|
| 71 | Left | Thalamus |
| 72 | Left | Caudate |
| 73 | Left | Putamen |
| 74 | Left | Pallidum |
| 75 | Left | Hippocampus |
| 76 | Left | Amygdala |
| 77 | Left | Accumbens nucleus |
| 78 | Right | Cerebellar white matter |
| 79 | Right | Cerebellum |
| 80 | Right | Thalamus |
| 81 | Right | Caudate |
| 82 | Right | Putamen |
| 83 | Right | Pallidum |
| 84 | Right | Hippocampus |
| 85 | Right | Amygdala |
| 86 | Right | Accumbens nucleus |
| 87 | - | Brainstem |

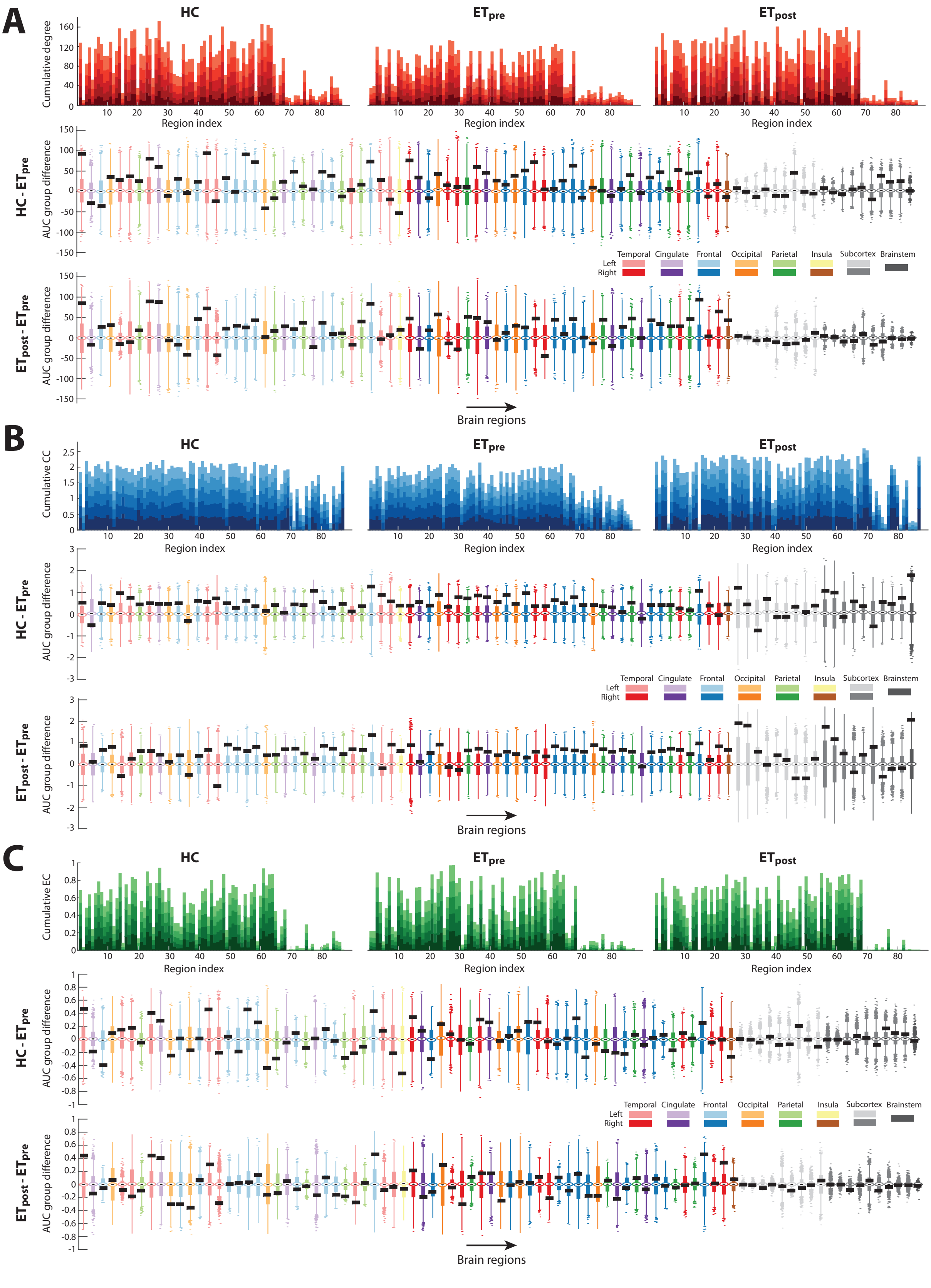


**Supplementary Figure 1. Graph theoretical analysis of cortical thickness.** For regional degree **(A)**, clustering coefficient **(B)** and eigenvector centrality **(C)**, regional values across edge densities from 20 to 60% (from dark to light colour shades for each stacked bar) in the HC (top left panel), $ET_{pre}$ (middle top panel) and $ET_{post}$ (top right panel) groups, as well as group differences for the HC - $ET_{pre}$ (middle panel) and $ET_{post}$ - $ET_{pre}$ (bottom panel) cases, where actual values are denoted by black rectangles and associated null distributions are reflected by box plots whose colour coding matches the brain lobe at hand.

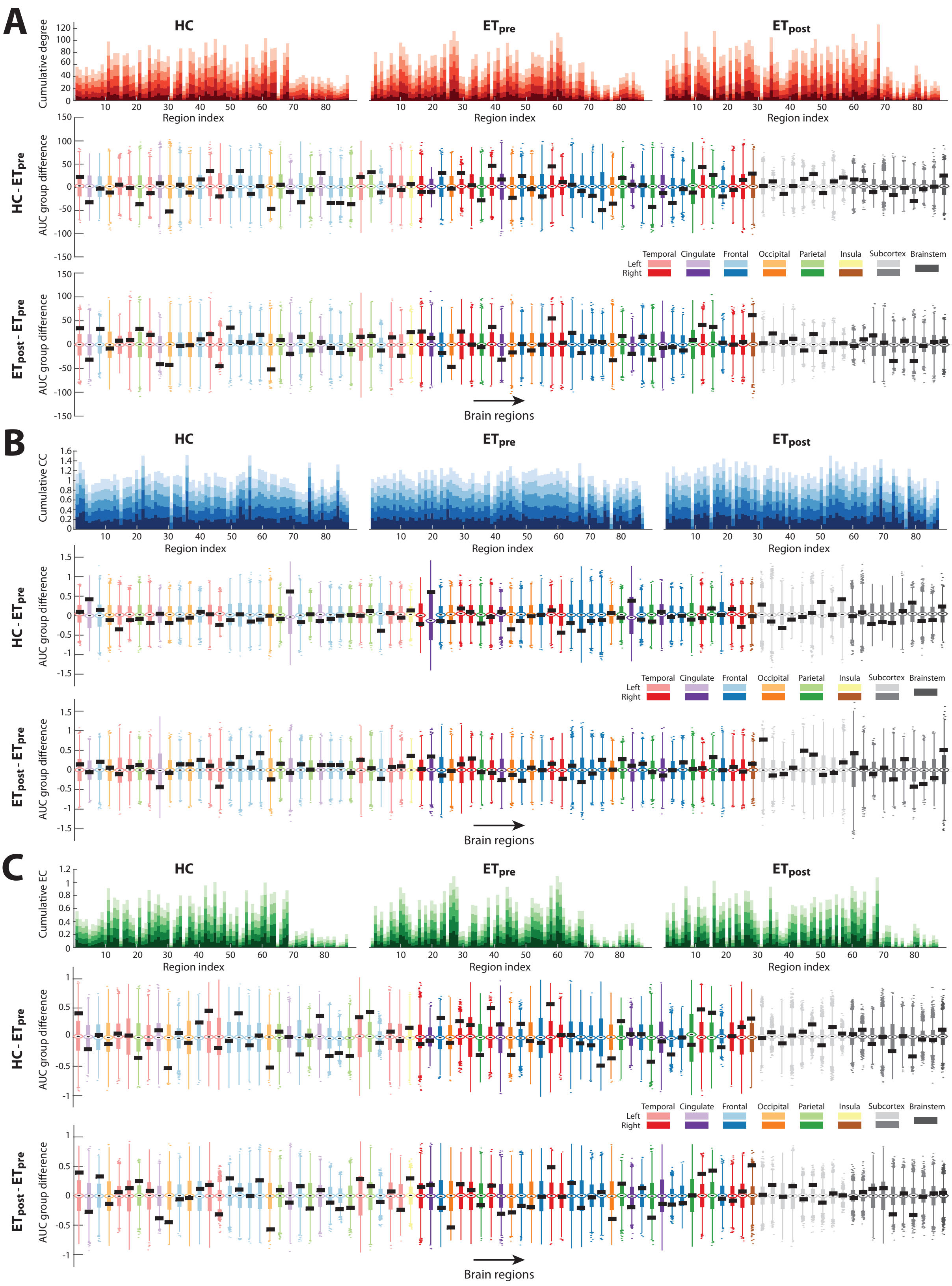


**Supplementary Figure 2. Graph theoretical analysis of surface area.** For regional degree **(A)**, clustering coefficient **(B)** and eigenvector centrality **(C)**, regional values across edge densities from 20 to 60% (from dark to light colour shades for each stacked bar) in the HC (top left panel), $ET_{pre}$ (middle top panel) and $ET_{post}$ (top right panel) groups, as well as group differences for the HC - $ET_{pre}$ (middle panel) and $ET_{post}$ - $ET_{pre}$ (bottom panel) cases, where actual values are denoted by black rectangles and associated null distributions are reflected by box plots whose colour coding matches the brain lobe at hand.

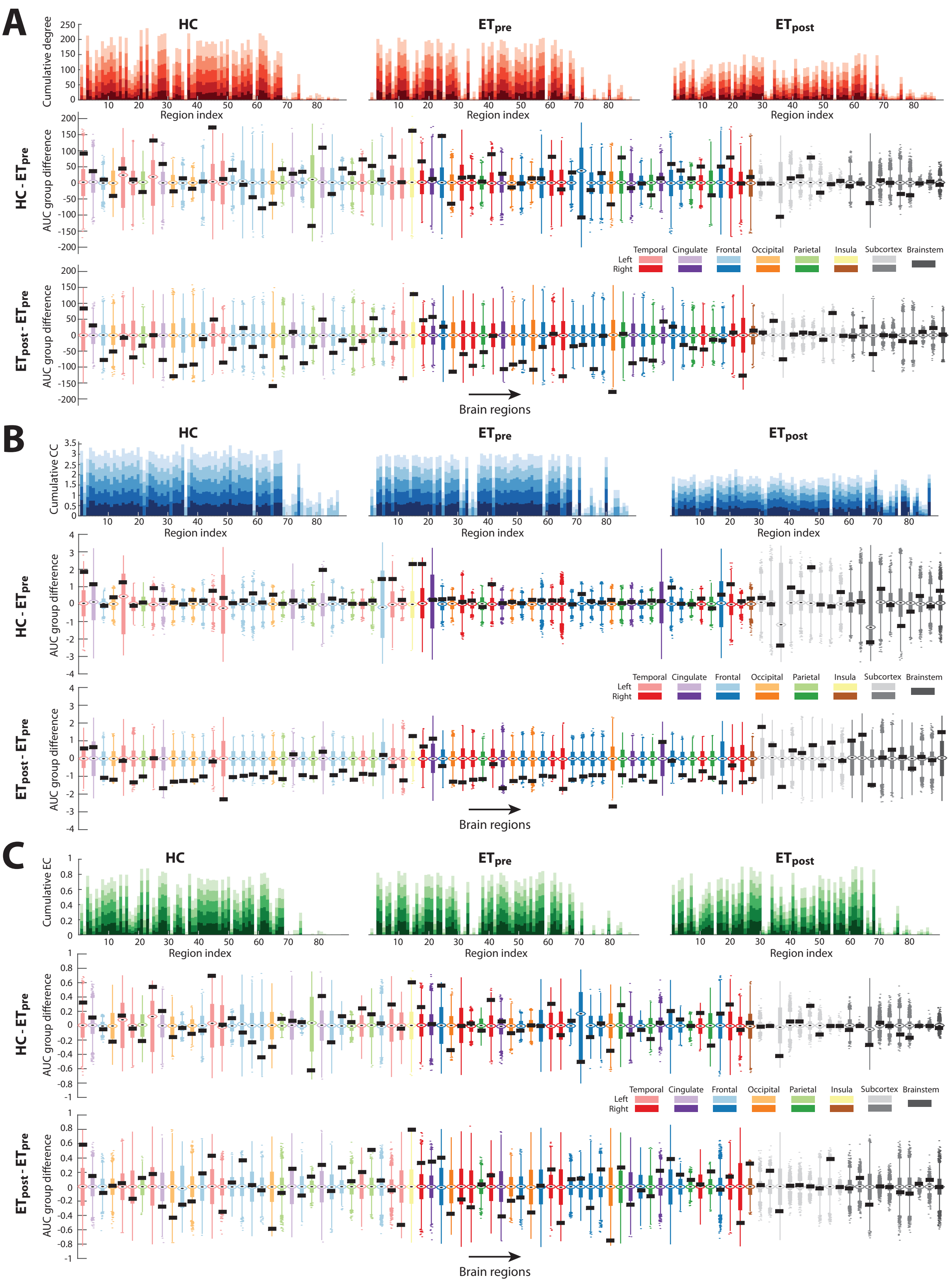


**Supplementary Figure 3. Graph theoretical analysis of mean curvature.** For regional degree **(A)**, clustering coefficient **(B)** and eigenvector centrality **(C)**, regional values across edge densities from 20 to 60% (from dark to light colour shades for each stacked bar) in the HC (top left panel), $ET_{pre}$ (middle top panel) and $ET_{post}$ (top right panel) groups, as well as group differences for the HC - $ET_{pre}$ (middle panel) and $ET_{post}$ - $ET_{pre}$ (bottom panel) cases, where actual values are denoted by black rectangles and associated null distributions are reflected by box plots whose colour coding matches the brain lobe at hand.

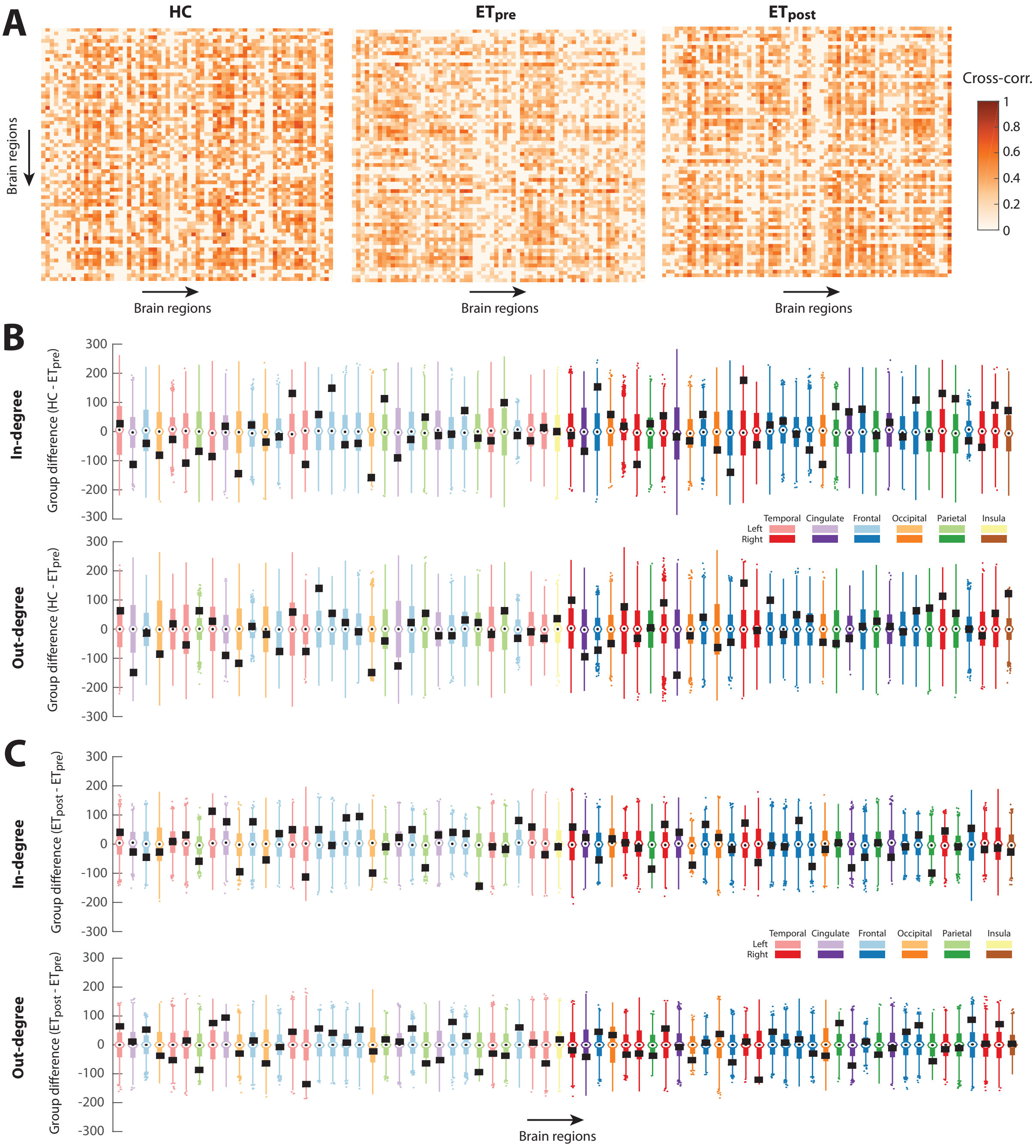


**Supplementary Figure 4. Graph theoretical analysis of cortical thickness/surface area interactions. (A)** For the HC (left), $ET_{pre}$ (middle) and $ET_{post}$ (right) groups, cross-correlation matrices reflecting the impact of cortical thickness change in a row region on surface area in a column one. **(B)** For the HC - $ET_{pre}$ group contrast, resulting regional in-degree (top panel) and out-degree (bottom panel) values across brain regions (left to right). Actual values are denoted by black squares, and associated null distributions with bar plots whose colour coding matches the brain lobe at hand. **(C)** Similar information for the $ET_{post}$ - $ET_{pre}$ group contrast.

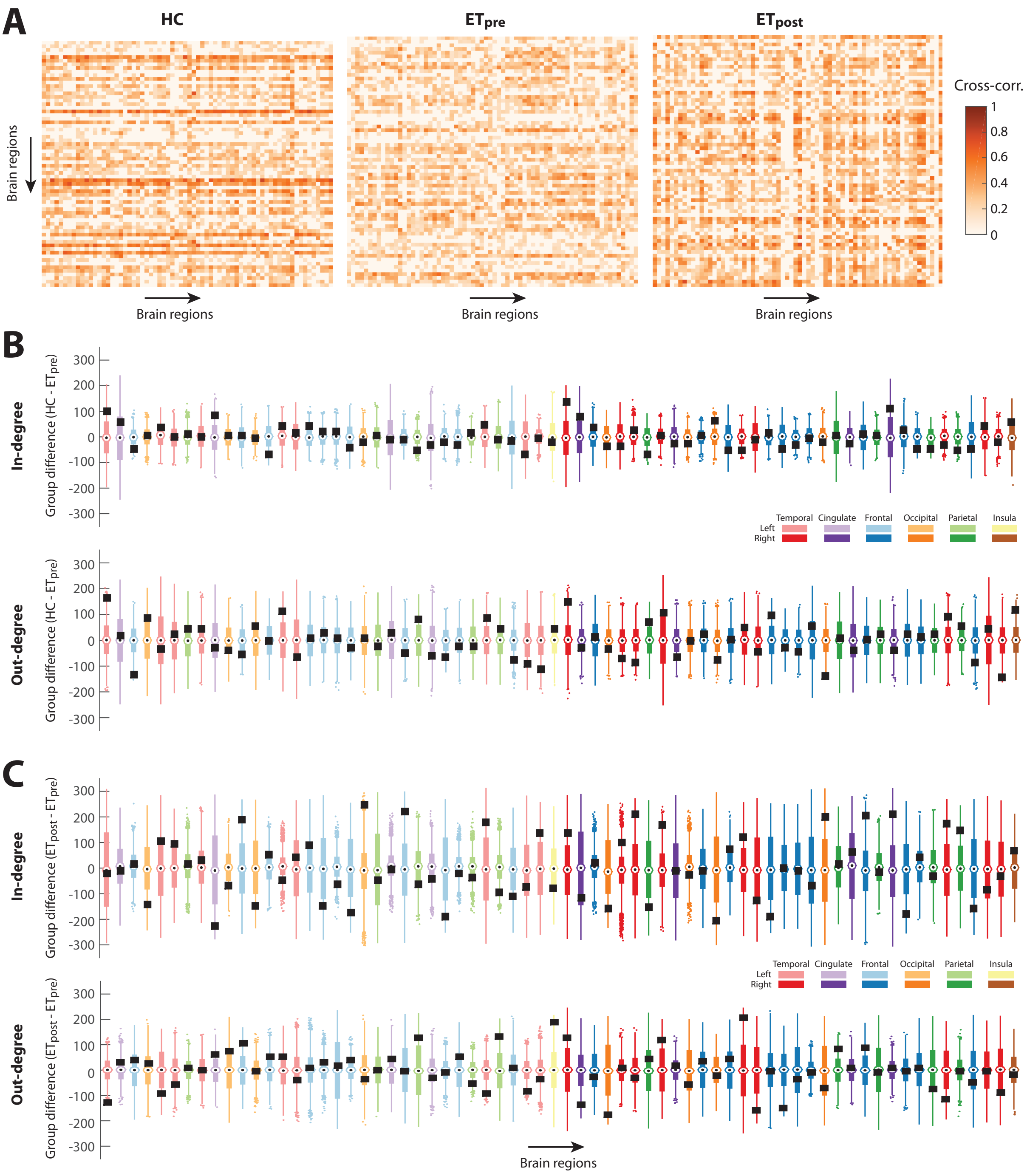


**Supplementary Figure 5. Graph theoretical analysis of surface area/mean curvature interactions. (A)** For the HC (left), $ET_{pre}$ (middle) and $ET_{post}$ (right) groups, cross-correlation matrices reflecting the impact of surface area change in a row region on mean curvature in a column one. **(B)** For the HC - $ET_{pre}$ group contrast, resulting regional in-degree (top panel) and out-degree (bottom panel) values across brain regions (left to right). Actual values are denoted by black squares, and associated null distributions with bar plots whose colour coding matches the brain lobe at hand. **(C)** Similar information for the $ET_{post}$ - $ET_{pre}$ group contrast.

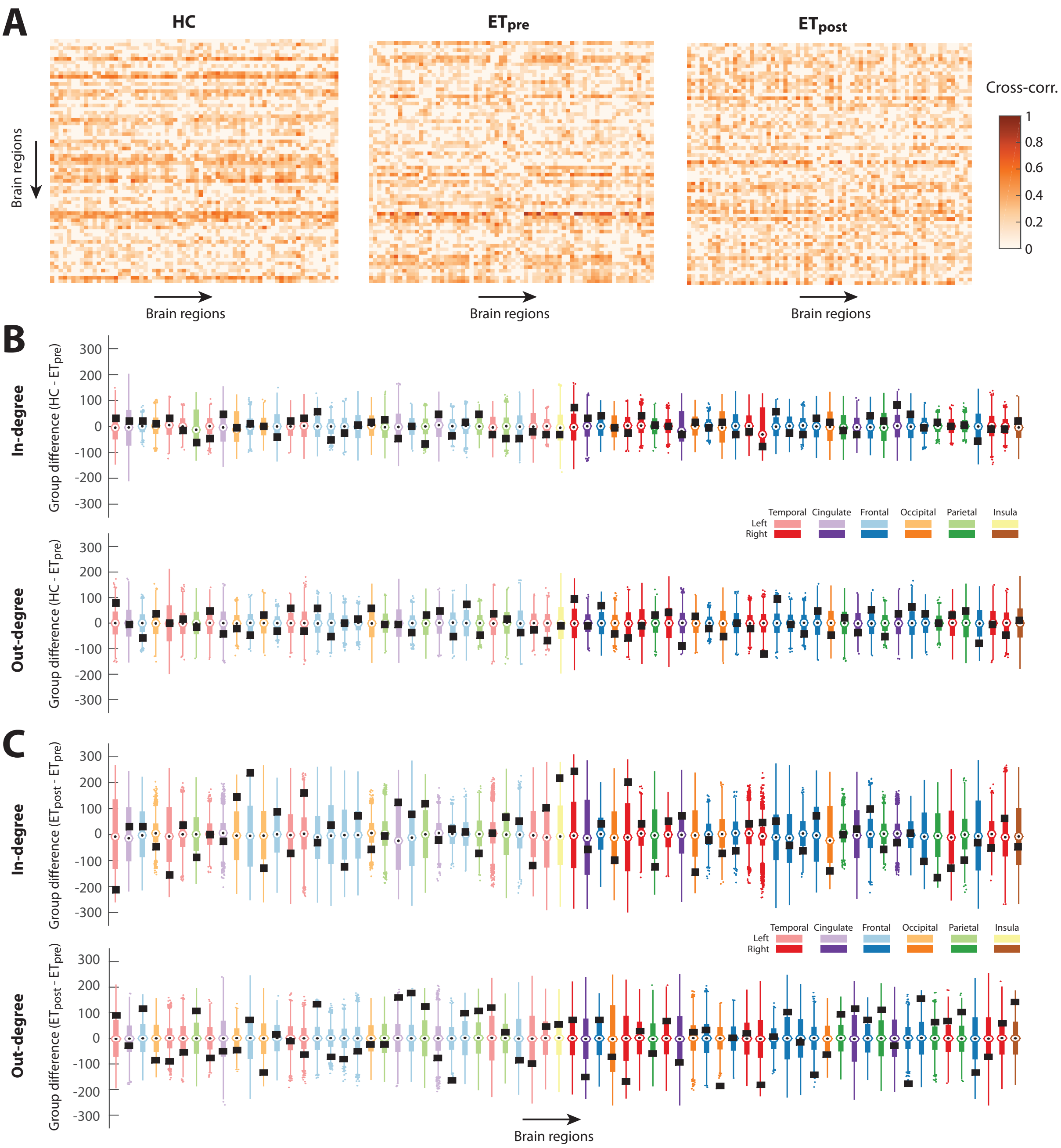


**Supplementary Figure 6. Graph theoretical analysis of cortical thickness/mean curvature interactions. (A)** For the HC (left), $ET_{pre}$ (middle) and $ET_{post}$ (right) groups, cross-correlation matrices reflecting the impact of cortical thickness change in a row region on mean curvature in a column one. **(B)** For the HC - $ET_{pre}$ group contrast, resulting regional in-degree (top panel) and out-degree (bottom panel) values across brain regions (left to right). Actual values are denoted by black squares, and associated null distributions with bar plots whose colour coding matches the brain lobe at hand. **(C)** Similar information for the $ET_{post}$ - $ET_{pre}$ group contrast.

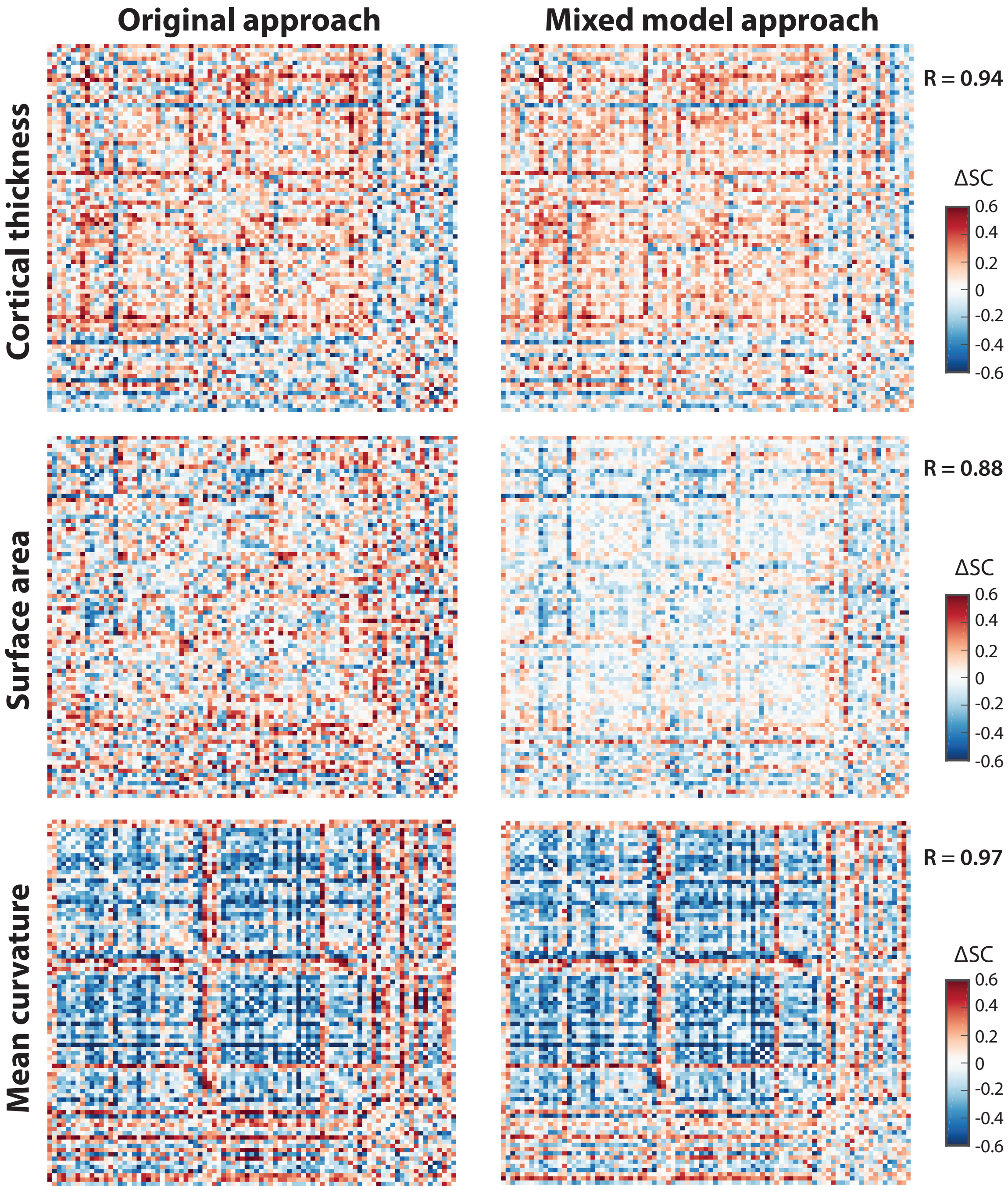


**Supplementary Figure 7. Comparison of $ET_{post}$ – $ET_{pre}$ structural covariance difference patterns across methods.** For the original approach based on group-wise structural covariance assessment using Pearson's correlation coefficient (left column), and for the mixed model approach (right column), whole-brain patterns of $ET_{post}$ – $ET_{pre}$ structural covariance differences for cortical thickness (top row), surface area (middle row) and mean curvature (bottom row). The spatial correlation *R* between the patterns extracted with both approaches is provided on top of the color bars.
